# Peripheral inflammation increases seizure susceptibility via the induction of neuroinflammation and oxidative stress in the hippocampus

**DOI:** 10.1186/s12929-015-0157-8

**Published:** 2015-06-24

**Authors:** Ying-Hao Ho, Yu-Te Lin, Chih-Wei J. Wu, Yung-Mei Chao, Alice Y. W. Chang, Julie Y. H. Chan

**Affiliations:** Department of Biological Sciences, National Sun Yat-sen University, Kaohsiung, 804 Taiwan; Division of Neurology, Kaohsiung Veterans General Hospital, Kaohsiung, 813 Taiwan; Center for Translational Research in Biomedical Sciences, Kaohsiung Chang Gung Memorial Hospital, Kaohsiung, 833 Taiwan; Department of Physiology and Institute of Basic Medical Sciences, National Cheng Kung University, Tainan, 701 Taiwan

**Keywords:** Systemic inflammation, Neuroinflammation, Pro-inflammatory cytokines, Microglia activation, Cycloxygenase-2, Oxidative stress, Hippocampus, Seizure

## Abstract

**Background:**

Neuroinflammation with activation of microglia and production of proinflammatory cytokines in the brain plays an active role in epileptic disorders. Brain oxidative stress has also been implicated in the pathogenesis of epilepsy. Damage in the hippocampus is associated with temporal lobe epilepsy, a common form of epilepsy in human. Peripheral inflammation may exacerbate neuroinflammation and brain oxidative stress. This study examined the impact of peripheral inflammation on seizure susceptibility and the involvement of neuroinflammation and oxidative stress in the hippocampus.

**Results:**

In male, adult Sprague-Dawley rats, peripheral inflammation was induced by the infusion of *Escherichia coli* lipopolysaccharide (LPS, 2.5 mg/kg/day) into the peritoneal cavity for 7 days via an osmotic minipump. Pharmacological agents were delivered via intracerebroventricular (i.c.v.) infusion with an osmotic minipump. The level of cytokine in plasma or hippocampus was analyzed by ELISA. Redox-related protein expression in hippocampus was evaluated by Western blot. Seizure susceptibility was tested by intraperitoneal (i.p.)  injection of kainic acid (KA, 10 mg/kg). We found that i.p. infusion of LPS for 7 days induced peripheral inflammation characterized by the increases in plasma levels of interleukin-1β (IL-1β), interleukin-6 (IL-6) and tumor necrosis factor-α (TNF-α). This is associated with a significant increase in number of the activated microglia (Iba-1^+^ cells), enhanced production of proinflammatory cytokines (including IL-1β, IL-6 and TNF-α), and tissue oxidative stress (upregulations of the NADPH oxidase subunits) in the hippocampus. These cellular and molecular responses to peripheral inflammation were notably blunted by i.c.v. infusion of a cycloxygenase-2 inhibitor, NS398 (5 μg/μl/h). The i.c.v. infusion of tempol (2.5 μg/μl/h), a reactive oxygen species scavenger, protected the hippocampus from oxidative damage with no apparent effect on microglia activation or cytokine production after peripheral inflammation. In the KA-induced seizure model, i.c.v. infusion of both NS398 and tempol ameliorated the increase in seizure susceptibility in animals succumbed to the LPS-induced peripheral inflammation.

**Conclusions:**

Together these results indicated that LPS-induced peripheral inflammation evoked neuroinflammation and the subsequent oxidative stress in the hippocampus, resulting in the increase in KA-induced seizure susceptibility. Moreover, protection from neuroinflammation and oxidative stress in the hippocampus exerted beneficial effect on seizure susceptibility following peripheral inflammation.

## Background

Status epilepticus is a common neurological emergency with considerable morbidity, mortality, and associated health-care costs. Emerging clinical and experimental evidence indicates that neuroinflammation with activated microglia and increased production of proinflammatory cytokines is involved in the pathophysiology of epilepsy [1–6]. Not only neuroinflammation is a shared feature of epileptic foci in the human brain and animal models [1–4], conditions that induce neuroinflammation and cytokine production facilitate seizure acquisition and epileptogenesis [5, 6]. Tissue oxidative stress, resultant from an imbalance between production over degradation of the reactive oxygen species (ROS), is another confounding factor in epilepsy [7, 8]. In the blood and surgically removed brain tissue of patients with epilepsy, oxidative stress markers, such as lipid peroxidation and activation of the nicotinamide adenine dinucleotide phosphate (NADPH) oxidase, are high and the antioxidant status is low [9, 10]. Furthermore, deficiency in brain antioxidant defense alone increases the severity of pentylenetetrazol- or kainic acid-induced seizures [11]. Conversely, treatment with antioxidants, including vitamin E [12] and vitamin C [13], protects the brain from oxidative stress-associated injury in epilepsy. While both neuroinflammation and brain oxidative stress are involved, relationship between these two factors in epileptogenesis, however, is not fully understood.

There is now compelling evidence that many neurological disorders, including seizure [14, 15], could be aggravated by peripheral inflammation. Several reports revealed that peripheral inflammation could induce neuroinflammation [16] via activation of microglia [17] and production of pro-inflammatory cytokines [18, 19], including interleukin-1β (IL-1β), interleukin-6 (IL-6), and tumor necrosis factor-α (TNF-α) in the brain. We reported previously that activation of microglia and production of cytokines in brain tissue after peripheral inflammation is cycloxygenase-2 (COX-2)-dependent [20]. The COX-2 enzyme is induced rapidly during seizures [21] and is distributed in the hippocampus [22]. However, whether the COX-2-dependent induction of neuroinflammation in the hippocampus plays a contributing role in the exacerbation of seizure susceptibility after peripheral inflammation is currently unknown.

A well-studied model of peripheral inflammation in rodents is systemic administration of *Escherichia coli* lipopolysaccharide (LPS) from Gram-negative bacteria to induce innate immune response [20, 23]. The present study took advantage of this model to investigate the significance of COX-2-dependent neuroinflammation in the hippocampus on seizure susceptibility following peripheral inflammation. Possible relationship between neuroinflammation and brain oxidative stress in the aggravated seizure susceptibility was also studied. Our results suggest that the LPS-induced peripheral inflammation evoked COX-2-dependent neuroinflammation and the subsequent oxidative stress in the hippocampus, resulting in the increase in seizure susceptibility. Moreover, cerebral application of COX-2 inhibitor or ROS scavenger protected the hippocampus from neuroinflammation and oxidative stress, and exerted beneficial effect on seizure susceptibility after peripheral inflammation.

## Methods

### Animals

Experiments were carried out in adult, male Sprague-Dawley rats (10-week-old, 250-282 g, *n* = 178) purchased from BioLASCO Taiwan Co., Ltd., Taipei, Taiwan. Animals were maintained under temperature control (24 ± 0.5 °C) and 12-h light-dark cycle (lights on between 08:00 and 20:00), and were housed in plastic cages with food and water available ad libitum. All experimental procedures were carried out in compliance with the guidelines of our institutional animal care and use committee. The minimal number of animals was used and care was taken to reduce any possible discomfort.

### Induction of chronic peripheral inflammation

Peripheral inflammation was induced by continuous infusion of *Escherichia coli* lipopolysaccharide (LPS, serotype 026:B6; Sigma, St. Louis, MO, USA) (2.5 mg/kg/day dissolved in saline) into the peritoneal cavity for 7 days via an osmotic minipump. On the day of pump implantation, animals were anesthetized with intraperitoneal (i.p.) injection of sodium pentobarbital (50 mg/kg) and an osmotic minipump (Alzet 1007D; Durect Co., Cupertino, CA, USA) was placed in the peritoneal cavity. Control animals received saline-filled osmotic minipumps. Animals received intramuscular procaine penicillin (1,000 IU) injection postoperatively, and only animals that showed progressive weight gain after the surgery were used in subsequent experiments. Protocol and dose of LPS infusion for induction of peripheral inflammation were modified from procedures reported previously [20].

### Intracerebroventricular infusion with an osmotic minipump

After implantation of osmotic pump into peritoneal cavity, some animals also received additional implantation of a micro-osmotic pump (model 1007D, ALZET) into the lateral ventricle with the aid of a brain infusion kit (brain infusion kit 2, ALZET) for intracerebroventricular (i.c.v.) infusion of the test agent. Procedures for implantation of the pump into the ventricle were adopted from previously published protocol [20]. Under the same anesthesia, animal was placed on the stereotaxic apparatus and a midline incision was made to expose dorsal surface of the skull. A burr hole was created at 0.9 mm posterior to the bregma and 1.5 mm lateral to the midline, and a cannula was inserted to the right lateral ventricle 4.0 mm below the pial surface. Drainage of cerebrospinal fluid (CSF) from the outlet of the cannula ensured patency of the implantation, which allows the test agent to be distributed to the entire ventricular system in both hemispheres. The cannula was sealed with dental cement and connected to an Alzet pump by medical grade vinyl tubing. The pump was placed subcutaneously in the dorsal back region. Test agents included NS398 (5 μg/μl/h dissolved in 1 % DMSO; Tocris Bioscience; Bristol, UK), tempol (2.5 μg/μl/h dissolved in saline; Merk KGaA, Darmstadt, Germany) or their corresponding vehicle. NS398 is a COX-2 inhibitor and is widely used as an anti-inflammatory agent [24]. Tempol scavenges the reactive oxygen species (ROS) and is a widely used antioxidant for study of oxidative stress [25]. Dose of the inhibitor was determined in our pilot experiments. At the end of each experiment, animals were killed with overdose of pentobarbital sodium (100 mg/kg, i.p.) to collect blood sample and hippocampus tissues for molecular, biochemical, and immunohistochemical analyses.

### Intraperitoneal administration of test agent

Some animals received peripheral treatment with NS398 (10 mg/kg; Tocris Bioscience) for 7 consecutive days beginning on the day of LPS infusion. This was carried out by a single bolus injection of the COX2 inhibitor into the peritoneal cavity. Daily injection was performed between 10:00-12:00 and the first injection was commended immediately after the implantation of the osmotic minipump for LPS infusion. Animals that received i.p. injection of 1 % DMSO served as vehicle control.

### Seizure susceptibility evaluation

In another separate series of experiments, seizure susceptibility was tested by i.p. injection of kainic acid (KA, 10 mg/kg). Animal behavior was monitored via video recording (HDR-XR260V, Sony, Tokyo, Japan) for 180 min to evaluate severity of seizure behaviors, using the Racine staging scale [26]: stage 0, normal; stage 1, immobilization, occasional “wet-dog shaking”; stage 2, head nodding, unilateral forelimb clonus, frequent “wet dog shaking”; stage 3, rearing, salivation, bilateral forelimb clonus; stage 4, generalized limbic seizures with falling, running and salivation; stage 5, continuous generalized seizures with tonic limbic extension. Total seizure activity was calculated by counting total stages of Racine scale in every 10 min for 180 min. Seizure onset time 3 (SOT3) was defined as the time (min) of seizure onset to Racine stage 3. Seizure susceptibility was evaluated by total seizure activity and SOT3.

### Plasma cytokine measurement

One milliliter of blood was collected from the heart and mixed with 1 mL Minicollect Wtripotassium EDTA (Greiner bio-one, Monroe, NC, USA) for quantitative analysis of IL-1β, IL-6, and TNF-α by the sandwich ELISA (Bender MedSystems, Burlingame, CA, USA) according to the manufacturer’s instructions. The colorimetric reaction product for individual proinflammatory factor was measured at 450 nm using a microplate reader (Dynex, Chantilly, VA, USA). The concentration of IL-1β, IL-6, or TNF-α, expressed in micrograms per milliliter (μg/mL), was determined from the regression line for the standards incubated under the same conditions in each assay. All assays were performed in triplicate.

### Hippocampal tissue collection

At the end of each experiment, rats were killed with an overdose of pentobarbital sodium (100 mg/kg, i.p.) and perfused intracardially with warm saline. The forebrain was rapidly removed and immediately frozen on dry ice. Hippocampal tissues from individual animal were collected and stored at -80 °C for subsequent protein analysis.

### Total protein isolation

Tissue samples from hippocampus were homogenized with a Dounce grinder with a tight pestle in ice-cold lysis buffer (15 mM HEPES, pH 7.2, 60 mM KCl, 10 mM NaCl, 15 mM MgCl2, 250 mM Sucrose, 1 mM EGTA, 5 mM EDTA, 1 mM PMSF, 2 mM NaF, 4 mM Na3VO4,). A mixture of leupeptin (8 μ/mL), aprotinin (10 μg/mL), phenylmethylsulfonyl fluoride (20 μg/mL), and trypsin inhibitor (10 μg/mL) was included in the isolation buffer to prevent protein degradation. The homogenate was centrifuged at 13,500 *g* for 10 min, and the supernatant was collected for protein analysis. The concentration of the total protein extracted was estimated by the method of Bradford with a protein assay kit (Bio-Rad, Hercules, CA, USA).

### Hippocampal cytokine measurement

Total protein extracted from each hippocampus was subject to quantitative analysis of IL-1β, IL-6 and TNF-α by the sandwich ELISA (Bender MedSystems) according to the same procedures described for plasma cytokine measurement. The concentration of cytokines was expressed as picogram per 100 μg total protein (pg/100 μg total protein). All assays were performed in triplicate.

### Detection of superoxide by electron paramagnetic resonance spectroscopy

ROS, in particular superoxide, production was measured by electron paramagnetic resonance (EPR) spectroscopy with hydroxylamine spin probe 1-hydroxy-3-carboxypyrrolidine (CPH), according to the previously published procedures [27]. Hippocampus homogenate was prepared and 20 μg of protein was added to 1 mM CPH and 0.1 mM diethylenetriaminepentaacetic acid in a total volume of 100 μL of Chelex-treated phosphate-buffered saline (PBS). Samples were placed in a 50 μL glass capillary (Wilmad Glass, Buena, NJ, USA). The EPR spectra were recorded using an EMX Plus EPR spectrometer (Bruker Biospin, Rheinstetten, Germany) equipped with an EMX-m40X microwave bridge operating at 9.87GHz. Data acquisition and processing were carried out using software provided by the manufacturer. Background signal was obtained by the omission of the spin trap; and was subtracted from the recorded EPR spectra for baseline correction.

### Western blot analysis

Total protein extracted from hippocampus was subject to Western blot analysis. The primary antisera used included goat polyclonal antiserum against  gp91^phox^ (1:5,000; BD Biosciences, Franklin Lakes, NJ, USA), p47^phox^ (1:5,000; Santa Cruz Inc., Dallus, TX, USA), p67^phox^ (1:5,000; Santa Cruz Inc.), p22^phox^ (1:5,000; Santa Cruz Inc.), copper/zinc SOD (Cu/ZnSOD, 1:3,000; Stressgen, Ann Arbor, MI, USA), manganese SOD (MnSOD, 1:6,000; Stressgen), catalase (1:4,000; Stressgen), or mouse monoclonal antiserum against glutathione peroxidase (GPx, 1:5,000; BD Biosciences) or rabbit polyclonal antiserum against Iba-1 (1:1,000; Wako, Tokyo, Japan). Membranes were washed with TBS-t buffer followed by incubation with horseradish peroxidase-conjugated goat anti-rabbit IgG or goat anti-mouse IgG (1:10,000; Jackson ImmunoReserach). Specific antibody-antigen complex was detected using an enhanced chemiluminescence Western blot detection system (GE Healthcare Bio-Sciences Corp., Piscataway, NJ, USA). Blotting bands were quantified by densitometry with Image J software (ImageJ 1.47v, NIH, USA). β-actin (1:20,000, Santa Cruz Inc.) was blotted on the same membrane as a loading control. The quantified values are expressed as a percentage to β-actin intensity.

### Immunohistochemistry

For immunohistochemical identification of the activated microglia cells, animals were perfused transcardially with 4 % paraformaldehyde in 0.1 M PBS (pH 7.4) under deep pentobarbital anesthesia (100 mg/kg, i.p.). The brain was removed and post-fixed overnight in the same fixative, followed by 30 % sucrose solution for at least 3 days. Coronal sections of the hippocampus at 25 μm were cut using a cryostat (Leica Microsystems, Wetzlar, Germany). After pre-absorption in gelatin (0.375 %), normal horse serum (3 %), and triton-X 100 (0.2 %) in PBS, the sections were incubated with a rabbit polyclonal antibody against Iba-1 (1:1,000; Wako), at room temperature overnight and then rinsed in PBS. After incubation in biotinylated horse anti-rabbit IgG (1:200; Jackson ImmunoResearch), the sections were rinsed in PBS and incubated with AB complexes (Vectastain ABC elite kit, Vector Laboratories, Burlingame, CA, USA). This was followed by washing the sections in PBS and incubated with a 3,3′-diaminobenzidine substrate kit (Vector Laboratories). Sections were counter stained with 1 % Neural Red, and observed under a light microscope (X51, Olympus, Tokyo, Japan).

### Statistical analysis

The values are expressed as means ± SEM. The statistical software SigmaStat (IBM SPSS Statistics 20.0, USA) was used for data analysis. One-way analysis of variance was used to assess group means, to be followed by the Scheffé multiple-range test for post hoc assessment of ndividual means. P <0.05 was considered to be statistically significant.

## Results

### Intraperitoneal LPS infusion induces peripheral inflammation

**Fig. 1:**
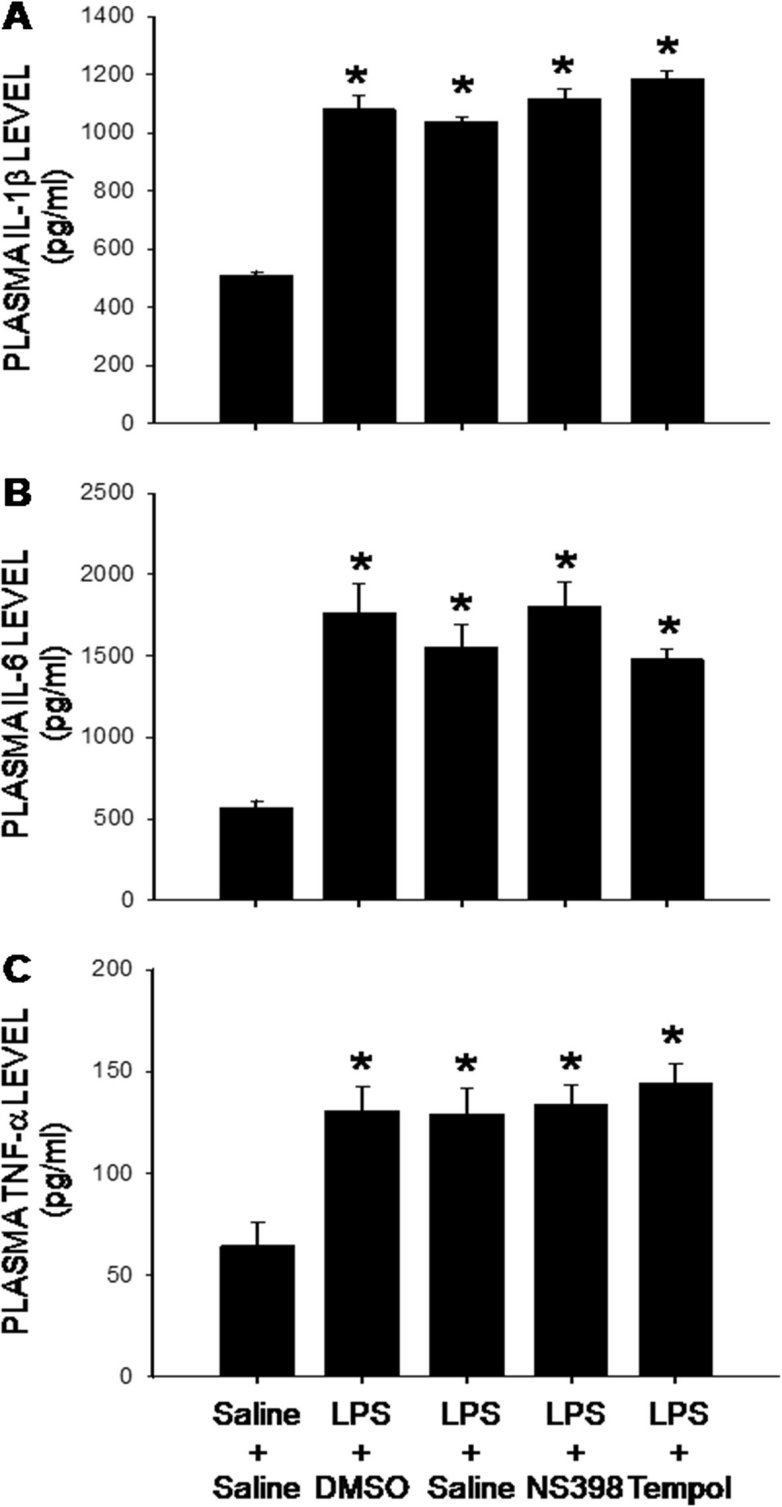
Intraperitoneal LPS infusion induces peripheral inflammation which is not affected by intracerebroventricular infusion of NS398 or tempol. Plasma levels of interleukin-1β (IL-1β, **a**), IL-6 (**b**), and tumor necrosis factor-α (TNF-α, **c**) measured on day 7 after intraperitoneal infusion via an osmotic minipump of saline or LPS (2.5 mg/kg/day) for 7 days alone or with additional intracerebroventricular infusion of NS398 (5 μg/μl/h, dissolved in 1 % DMSO), tempol (2.5 μg/μl/h, dissolved in saline), or the corresponding vehicle. Values are mean ± SEM, *n* = 10–12 rats per group. *P <0.05 vs. saline-treated group in the post hoc Scheffé multiple-range test

Compared to saline, infusion into the peritoneal cavity of LPS (2.5 mg/kg/day) for 7 days significantly increased plasma levels of IL-1β, IL-6 and TNF-α (Fig. 1). The LPS-induced peripheral inflammation was significantly attenuated by daily i.p. injection of a COX-2 inhibitor, NS398 (10 mg/kg; solved in 1 % DMSO) (Table 1), but not by concurrent i.c.v. infusion of NS398 (5 μg/μl/h), or a ROS scavenger, tempol (2.5 μg/μl/h) for 7 days (Fig. 1). On day 7 after peripheral infusion of the endotoxin, there was no significant change in body weight (262 ± 5 vs 257 ± 4 g, *P* > 0.05, *n* = 8 in each group), body temperature (36.4 ± 1.2 vs 37.3 ± 2.1 °C, *P* > 0.05, *n* = 8 in each group, daily food (24 ± 4 vs 21 ± 5 g/day, *P* > 0.05, *n* = 8 in each group) or water (43 ± 6 vs 46 ± 5 mL/day, *P* > 0.05, *n* = 8 in each group) intake between the saline- and LPS-treated animals.

**Table 1:** Effect of daily bolus injection of NS398 (10 mg/kg, i.p.) on the increases in plasma proinflammatory cytokine levels following intraperitoneal infusion of *Escherichia coli* lipopolysaccharide (LPS, 2.5 mg/kg/day) for 7 days

Cytokines	Treatment			
(pg/ml)	Saline	LPS	LPS + DMSO	LPS + NS398
IL-1β	485 ± 62	1086 ± 135^*^	1226 ± 165^*^	637 ± 88^**^
IL-6	567 ± 83	1669 ± 231^*^	1450 ± 204^*^	709 ± 74^**^
TNF-α	68 ± 11	127 ± 22^*^	136 ± 24^*^	75 ± 18^**^

Data are mean ± S.E.M., *n* = 6-8 animals per group

^*^ *P* < 0.05 versus saline group; ^**^ *P* < 0.05 versus LPS group in the Scheffé multiple-range test

### Peripheral LPS infusion induces a COX-2-dependent neuroinflammation in the hippocampus

**Fig. 2:**
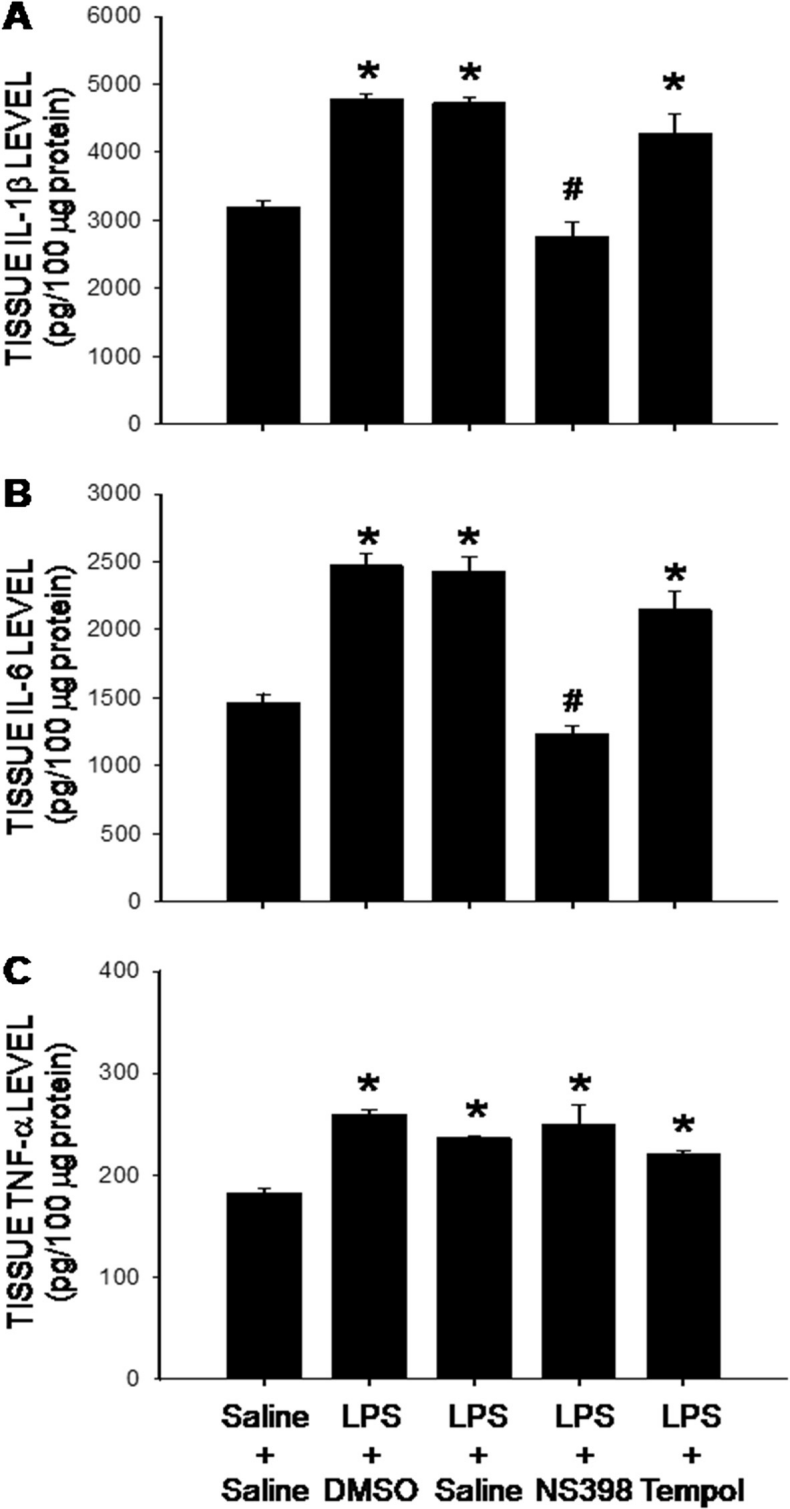
Intraperitoneal LPS infusion induces a COX-2-dependent neuroinflammation in the hippocampus. Tissue levels of IL-1β (**a**), IL-6 (**b**) and TNF-α (**c**) in hippocampus measured on 7 day after intraperitoneal infusion via an osmotic minipump of saline or LPS (2.5 mg/kg/day) for 7 days alone or with additional intracerebroventricular infusion of NS398 (5 μg/μl/h, dissolved in 1 % DMSO), tempol (2.5 μg/μl/h, dissolved in saline), or the corresponding vehicle. Values are mean ± SEM, *n* = 10–12 rats per group. *P <0.05 vs. saline-treated group; ^#^P <0.05 vs. corresponding LPS-treated group in the post hoc Scheffé multiple-range test

In addition to the induction of peripheral inflammation, i.p. infusion of LPS (2.5 mg/kg/day) for 7 days also induced neuroinflammation in the hippocampus, characterized by the increases in tissue levels of IL-1β, IL-6 and TNF-α (Fig. 2). Interestingly, the peripheral LPS-induced increases in IL-1β and IL-6, but not TNF-α, production in the hippocampus were appreciably blunted by a concomitant infusion into the lateral ventricle of NS398 (5 μg/μl/h), but not tempol (2.5 μg/μl/h). In addition, daily i.p. injection of NS398 (10 mg/kg) for 7 days also significantly reversed the hippocampal neuroinflammation after the LPS infusion (Table 2).

**Table 2:** Effect of daily bolus injection of NS398 (10 mg/kg, i.p.) on the increases in tissue proinflammatory cytokine levels in the hippocampus following intraperitoneal infusion of *Escherichia coli* lipopolysaccharide (LPS, 2.5 mg/kg/day) for 7 days

Cytokines	Treatment			
(pg/100 μg protein)	Saline	LPS	LPS + DMSO	LPS + NS398
IL-1β	968 ± 79	1224 ± 106^*^	1145 ± 154^*^	868 ± 91^**^
IL-6	365 ± 45	577 ± 72^*^	613 ± 98^*^	416 ± 65^**^
TNF-α	191 ± 24	272 ± 38^*^	259 ± 36^*^	180 ± 28^**^

Data are mean ± S.E.M., *n* = 6-8 animals per group

^*^ *P* < 0.05 versus saline group; ^**^ *P* < 0.05 versus LPS group in the Scheffé multiple-range test

### Peripheral LPS infusion induces a COX-2 dependent increase in superoxide production in the hippocampus

**Fig. 3:**
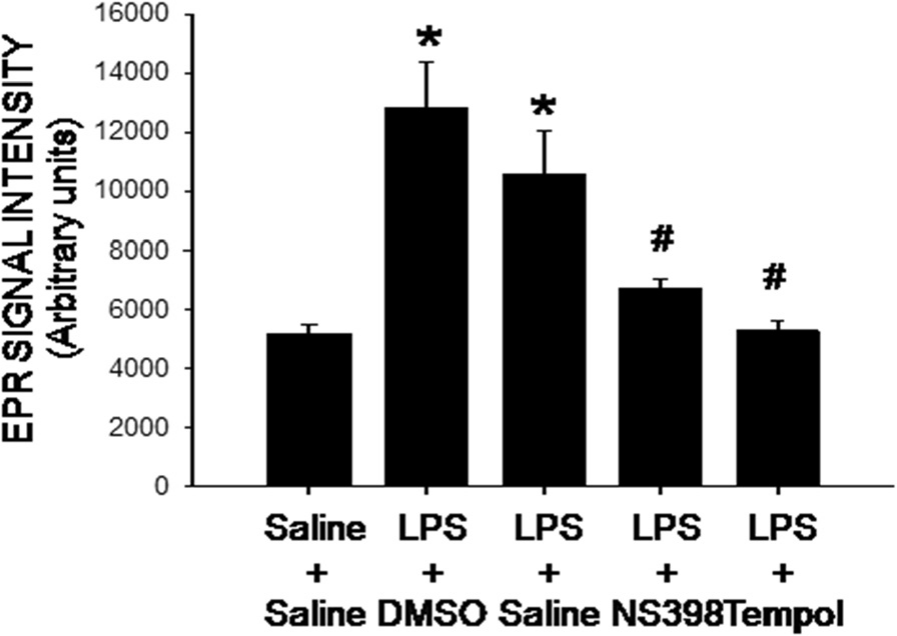
Intraperitoneal LPS infusion induces a COX-2-dependent and redox-sensitive increase in tissue superoxide levels in the hippocampus. Tissue level of superoxide anion, measured by electron paramagnetic resonance (EPR) spectroscopy, in the hippocampus on day 7 after intraperitoneal infusion via an osmotic minipump of saline or LPS (2.5 mg/kg/day) for 7 days alone with additional intracerebroventricular infusion NS398 (5 μg/μl/h, dissolved in 1% DMSO), tempol (2.5 μg/μl/h, dissolved in saline) or the corresponding vehicle. The concentration of superoxide in arbitrary unit was derived from computer simulation of the EPR spectra by normalized line shapes. Values are mean ± SEM, *n* = 8–10 rats per group. *P <0.05 vs. saline-treated group; ^#^P <0.05 vs. LPS-treated group in the post hoc Scheffé multiple-range test

Peripheral inflammation may exacerbate brain oxidative stress [20]. On day 7 after peripheral LPS (2.5 mg/kg/day) infusion, tissue level of ROS, in particular superoxide, in the hippocampus was significantly increased, compared to the saline-treated groups (Fig. 3). This induced increase in tissue superoxide level in the hippocampus was abolished by i.c.v. infusion of NS398 (5 μg/μl/h) or tempol (2.5 μg/μl/h) for 7 days.

### The COX-2-dependent upregulations of the NADPH oxidase subunit and antioxidant proteins in the hippocampus following peripheral inflammation

**Fig. 4:**
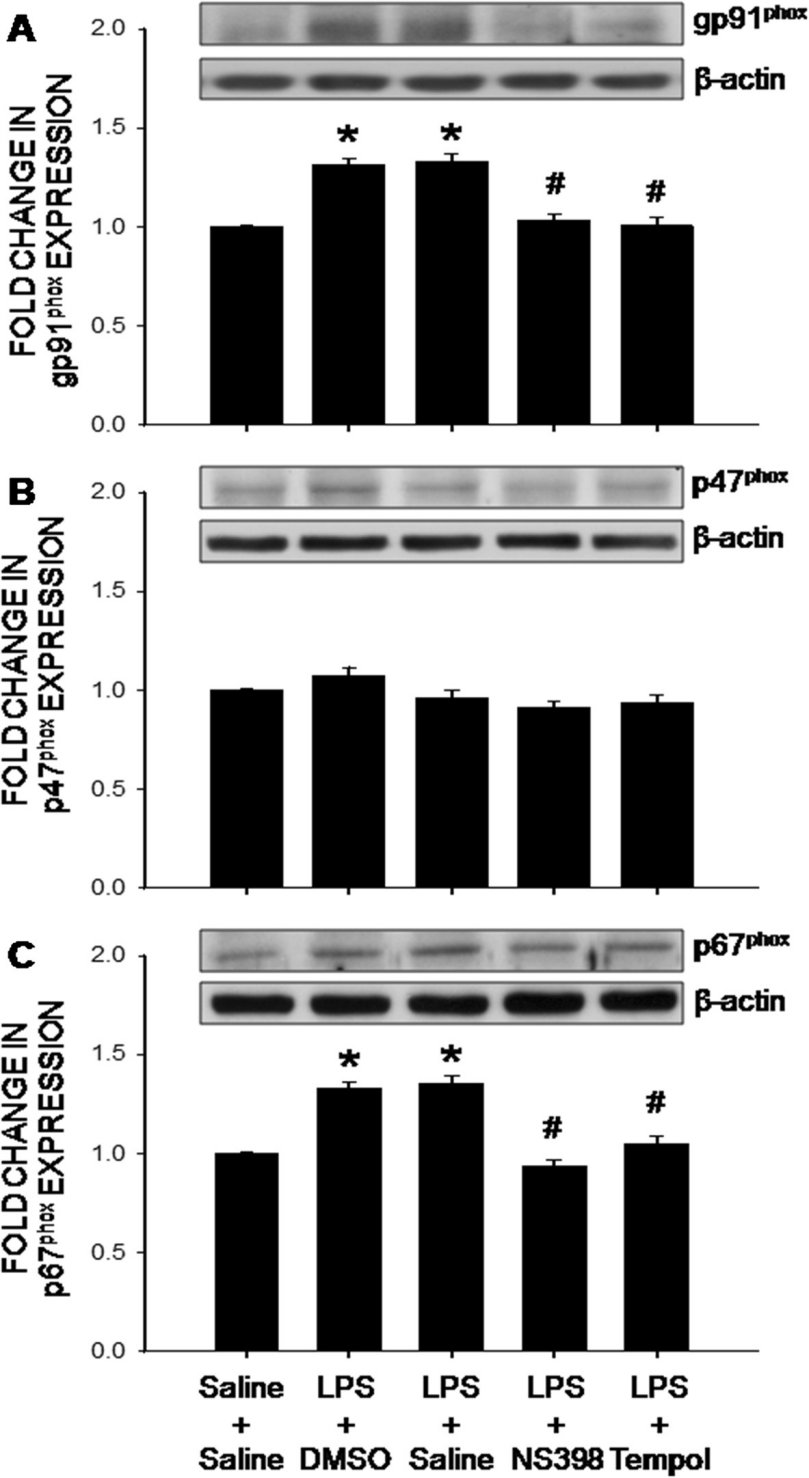
The COX-2-dependent upregulations of the NADPH oxidase subunits in the hippocampus after intraperitoneal LPS infusion. Representative gels (insert) and densitometric analysis of results from Western blot showing changes in expression of gp91^phox^ (**a**), p47^phox^ (**b**) and p67^phox^ (**c**) in the hippocampus, measured on day 7 after intraperitoneal infusion via an osmotic minipump of saline or LPS (2.5 mg/kg/day) for 7 days alone or with additional intracerebroventricular infusion of NS398 (5 μg/μl/h, dissolved in 1 % DMSO), tempol (2.5 μg/μl/h, dissolved in saline) or the corresponding vehicle. Values are mean ± SEM, *n* = 8–10 rats per group. *P <0.05 vs. saline-treated group; ^#^P <0.05 vs. LPS-treated group in the post hoc Scheffé multiple-range test

**Fig. 5:**
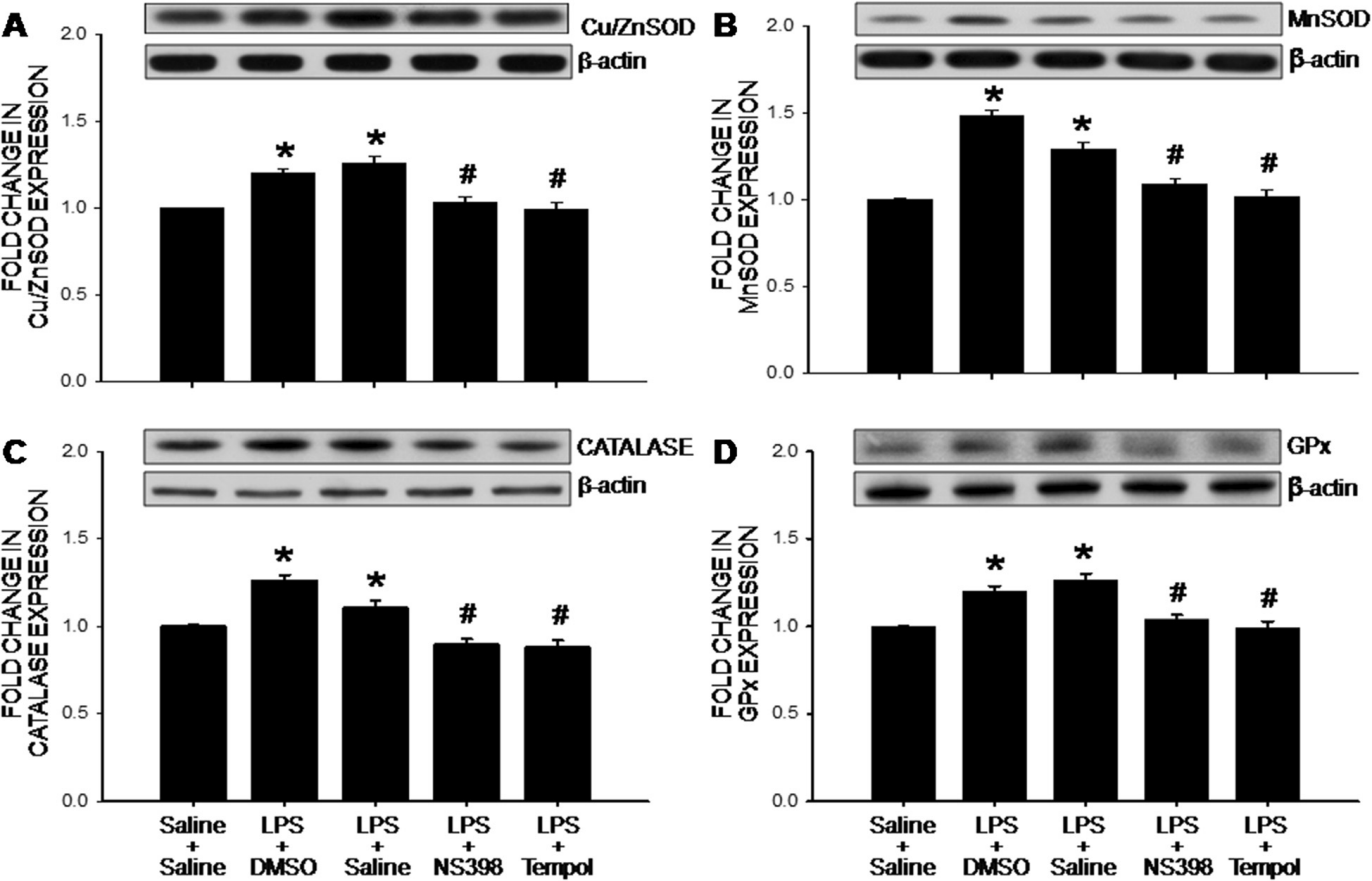
The COX-2-dependent upregulations of antioxidant proteins in the hippocampus after intraperitoneal LPS infusion. Representative gels (insert) and densitometric analysis of results from Western blot showing changes in expression of cupper/zinc superoxide dismutase (Cu/ZnSOD) (**a**), manganese SOD (MnSOD) (**b**), catalase (**c**), or glutathione peroxide (GPx) (**d**) in hippocampus, measured on day 7 after intraperitoneal infusion via an osmotic minipump of saline or LPS (2.5 mg/kg/day) for 7 days alone or with additional intracerebroventricular infusion of NS398 (5 μg/μl/h, dissolved in 1 % DMSO), tempol (2.5 μg/μl/h, dissolved in saline) or the corresponding vehicle. Values are mean ± SEM, *n* = 8–10 rats per group. *P <0.05 vs. saline-treated group; ^#^P <0.05 vs. LPS-treated group in the post hoc Scheffé multiple-range test

Accumulation of ROS in the hippocampus indicates an imbalance in tissue redox homeostasis. At the end of 7-day peripheral LPS infusion, protein expressions of the gp91^phox^ and p67^phox^, but not p47^phox^, subunits of the NADPH oxidase in the hippocampus were significantly increased (Fig. 4). At the same time, protein expression of antioxidants, including Cu/Zn SOD, MnSOD, catalase and GPx, were also increased in the hippocampus (Fig. 5). All these molecular events induced by peripheral LPS infusion were notably attenuated by i.c.v. infusion of NS398 (5 μg/μl/h) or tempol (2.5 μg/μl/h) for 7 days. Protein expression of p22^phox^ subunit in the hippocampus was under our detection limit; and was not affected by peripheral LPS infusion alone or with additional i.c.v. infusion of the inhibitors (data not shown).

### Peripheral LPS infusion induces a COX-2-dependent microglial activation in the hippocampus

**Fig. 6:**
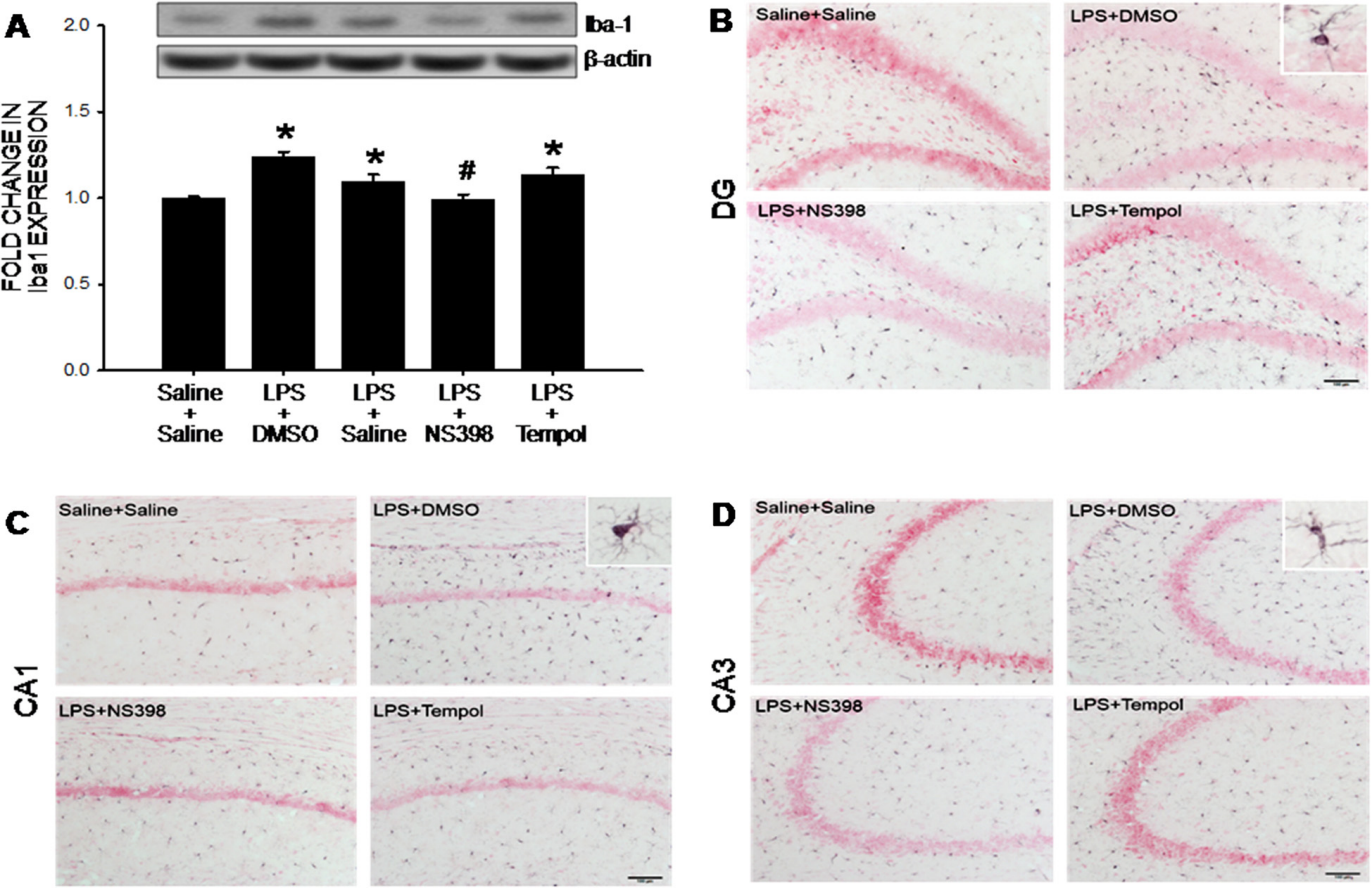
Intraperitoneal LPS infusion induces a COX-2-dependent microglial activation in the hippocampus. Gels (inset) and densitometric analysis of results from Western blot showing changes in the expression of Iba-1 (**a**) or representative photomicrographs (**b**-**d**) showing immunoreactivity to Iba-1 in hippocampal area (CA1, CA3, and dentate gyrus, DG), measured on day 7 after intraperitoneal infusion via an osmotic minipump of saline or LPS (2.5 mg/kg/day) for 7 days alone or with additional intracerebroventricular infusion of NS398 (5 μg/μl/h, dissolved in 1 % DMSO), tempol (2.5 μg/μl/h, dissolved in saline) or the corresponding vehicle. Also shown are photomicrographs of a typical activated microglia in a higher magnification (insets in **b**-**d**). Values are mean ± SEM, *n* = 8–10 rats per group. *P <0.05 vs. saline-treated group; ^#^P <0.05 vs. LPS-treated group in the post hoc Scheffé multiple-range test. Scale bar in (**b**-**d**): 100 μm

Microglial activation plays an active role in the induction of neuroinflammation. Compared to saline infusion, peripheral LPS infusion resulted in a significant increase in protein expression of Iba-1, an experimental index for activated microglia (Fig. 6a). Immunohistochemical analysis further demonstrated that the Iba-1-positive cells were distributed in the CA1, CA3 and dentate gyrus (DG) of the hippocampus (Fig. 6b-d); and was notably attenuated by concurrent i.c.v. infusion of NS398 (5 μg/μl/h), but not tempol (2.5 μg/μl/h) infusion.

### COX-2-dependent and redox sensitive increase in seizure susceptibility following peripheral inflammation

**Fig. 7:**
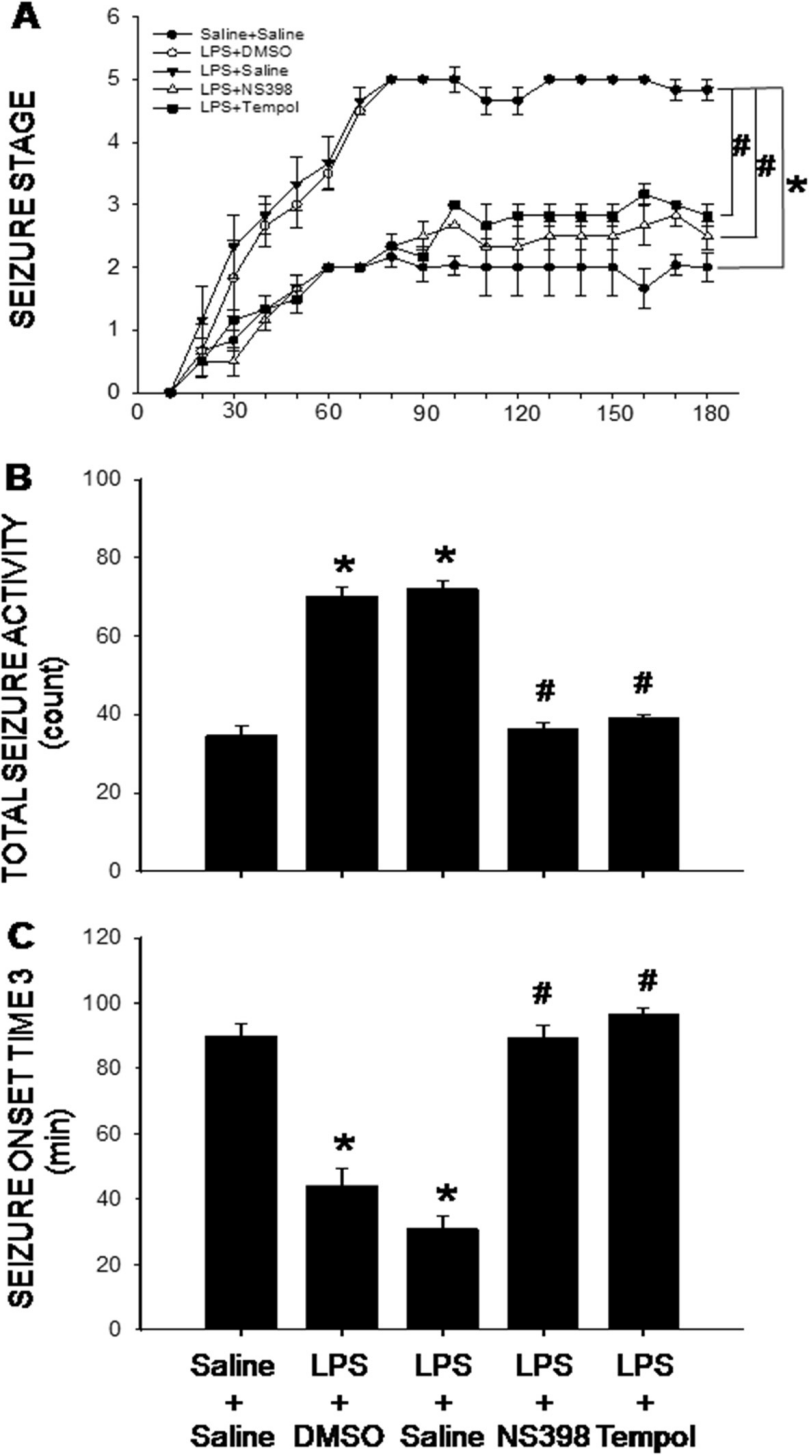
COX-2-dependent and redox-sensitive increase in seizure susceptibility after intraperitoneal LPS infusion. Time-course changes in kainic acid-induced seizure stage (a), total seizure activity (stages) (**b**) and seizure onset time to stage 3 (**c**) measured on day 7 after intraperitoneal infusion via an osmotic minipump of saline or LPS (2.5 mg/kg/day) for 7 days alone or with additional intracerebroventricular infusion of NS398 (5 μg/μl/h, dissolved in 1 % DMSO), tempol (2.5 μg/μl/h, dissolved in saline) or the corresponding vehicle. Values are mean ± SEM, *n* = 8-10 rats per group. *P <0.05 vs. saline-treated group; ^#^P <0.05 vs. LPS-treated group in the post hoc Scheffé multiple-range test

Seizure susceptibility was tested by a singly bolus injection into the peritoneal cavity of KA (10 mg/kg). On day 7 after the LPS infusion, seizure susceptibility (Fig. 7a), based on the Racine staging scale evaluation, was significantly increased; alongside the increase in total seizure activity (Fig. 7b) and decrease in seizure onset time to stage 3 (Fig. 7c). Changes in all these parameters were blunted by i.c.v. infusion of NS398 (5 μg/μl/h) or tempol (2.5 μg/μl/h). Brain infusion of NS398 or tempol alone had no discernible effect on seizure activity in animals subjected to peripheral infusion of saline (data not shown).

## Discussion

Peripheral inflammation, a shared feature of many diseases, such as rheumatoid arthritis, inflammatory bowel disease, sepsis, and inflammatory lung disease [28, 29], is capable to exacerbate neurological disorders, including seizure [14, 30]. In the present study we have shown that in a rodent model of endotoxin-induced peripheral inflammation, the seizure susceptibility is significantly increased; alongside the activation of microglia, induction of neuroinflammation and tissue oxidative stress in the hippocampus. Moreover, treating the injured hippocampus with a COX-2 inhibitor mitigates microglia activation, ameliorates the inflammatory conditions and protects the tissue from oxidative stress. At the same time, the inhibitor reduces seizure susceptibility in animal succumbed to peripheral inflammation. Together our results suggest that peripheral inflammation activates microglia and induces a COX-2-dependent neuroinflammation and oxidative stress in hippocampus, leading to the increase in KA-induced seizure susceptibility.

Systemic infusion of the Gram-negative bacterial endotoxin is a well-characterized rodent model of peripheral inflammation [20, 23]. In this animal model, we found that the LPS-induced peripheral inflammation is associated with the appearance of an inflammatory response in the hippocampus similar to that generated in the periphery. This is evident by activation of microglia and the increases in production of proinflammatory cytokines, including IL-1β, IL-6 and TNF-α. Given that i.p. injection of an effective dose of NS398 which blocks plasma cytokine production (cf. Table 1) could significantly diminish neuroinflammation in the hippocampus (cf. Table 2), it is unlikely that hippocampal neuroinflammation is a direct effect of LPS to the brain tissue. Instead, these data strongly suggest that peripheral inflammation induced by the LPS triggers a COX-2-dependent mechanism to induce neuroinflammation in the hippocampus. Several mechanisms have been proposed for the peripheral inflammation to induce a mirror inflammation in the brain. These include the expression of toll-like and IL receptors in circumventricular organs of the brain [31], communication by the vagal afferents [32], microglial activation [33] and potential disruption of the blood-brain barrier [34]. We reported previously that the blood-brain barrier is reasonably well preserved following peripheral LPS infusion [20]. It is conceivable that the observed neuroinflammation in hippocampus may not result from the direct entry of blood-borne inflammatory cytokines to this neural substrate. We noted that in contrast to IL-1β and IL-6, i.c.v. infusion of NS398 has no effect in blocking TNF-α production, indicating differential mechanisms might underlie cytokine production in the hippocampus after peripheral inflammation.

Neuroinflammation has long been recognized as an important factor in seizure pathophysiology [35]. We found in the present study that augmented cytokine production and microglial activation in the hippocampus appear necessary and sufficient for increase in seizure susceptibility after peripheral inflammation. Two lines of evidence support this notion. First, there is a close correlation in microglial activation and the increases in pro-inflammatory cytokine production in the hippocampus to the increase in seizure susceptibility in animal succumbed to peripheral inflammation. Moreover, NS398 in a dose that effectively blocks microglial activation and pro-inflammatory cytokine production in the hippocampus also diminishes the increase in seizure susceptibility after peripheral inflammation. We noted that the dose of NS398 which reduces hippocampal cytokine production (cf. Fig. 2) has no effect on LPS-induced plasma inflammation (cf. Fig. 1). These results further deem unlikely the increase in seizure susceptibility a direct consequence to peripheral inflammation and support the notion of hippocampal neuroinflammation in aggravation of seizure susceptibility. Cellular and molecular events mediating the hippocampal COX-2-dependent and neuroinflammation-associated increase in seizure susceptibility after peripheral inflammation await further elucidation. To this end, generation of a functional caspase-1-containing inflammasome in driving the proinflammatory programmed cell death, termed “pyroptotic death” [36], in the hippocampus has recently been demonstrated to play a key role in the epileptogenic process [37]. In addition, COX-2-dependent microglial activation in the hippocampus was proposed to enhance glutamatergic synaptic transmission and synaptic currents in the hippocampus during peripheral organ inflammation [38]. The elevated glutamatergic synaptic transmission in the hippocampus is involved in prolonged epileptiform discharges [39].

There is a growing body of evidence showing the disturbance of cellular redox homeostasis and the increase in ROS production in patients with epilepsy [40, 41]. Based on quantification of tissue ROS by EPR spectroscopy, we found that superoxide level in the hippocampus was significantly increased after peripheral inflammation. Our results further indicate that such an increase may result from the increases in protein expression of gp91^phox^ and p67^phox^ subunits of the NADPH oxidase. The increase in gp91^phox^ protein expression is in agreement with a previous report showing an upregulation of gp91^phox^ (*Cybb*) gene expression in the hippocampus after systemic LPS administration [42]. Moreover, the gp91^phox^ subunit was proposed to be the main source of ROS involved in oxidative damage to the hippocampus in the cecal ligation and puncture model of sepsis [43]. In contrast, our results are in difference to those by Czapski et al. showing upregulations of p47^phox^ and p22^phox^ subunit mRNA to acute systemic inflammation. At day 7 after systemic LPS infusion, we found protein level of p47^phox^ was slightly, albeit insignificantly, affected by the endotoxin; whereas p22^phox^ subunit in the hippocampus was below our detection limit. We reason the discrepancy might be related to duration (acute versus long-term) and animal model (mice versus mouse) of peripheral inflammation. Our results on the expression of antioxidants further revealed the increases in major antioxidants, including SODs, catalase and GPx, in the hippocampus after peripheral inflammation. Given that the increases in these antioxidants are inhibited by the i.c.v. treatment of tempol (cf. Fig. 5), it is likely that these molecular events are consequences to, rather than the cause of, tissue oxidative stress. Previous studies have reported that generation of ROS in hippocampus [44, 45] because of an upregulation of NADPH oxidase [9, 46, 47], SOD [48–50], catalase [51] and GPx [48] is associated with seizure. Together, we postulate that after peripheral inflammation, the COX-2-dependent increase in NADPH oxidase expression evokes superoxide production and ROS accumulation in the hippocampus, which in turn activates the endogenous antioxidant defense signals in an attempt to degrade the newly produced superoxide and to mitigate oxidative stress. However, under a sustained peripheral inflammation, the COX-2-dependent and NADPH oxidase-associated ROS production may exceed endogenous antioxidant defenses, resulting in an accumulation of ROS and tissue oxidative stress in the hippocampus. Since we did not measure enzyme activity in the present study, possibility that oxidative stress in hippocampus the result of neuroinflammation-induced inhibition of antioxidant enzyme activities, despite the increases in their protein expressions, could not be excluded. We realize that in addition to the NADPH oxidase, mitochondria are another major source for cellular ROS [52]. In this regard, systemic injection of LPS induces region specific mitochondrial dysfunction in the hippocampus [53]. In addition, a selective dysfunction of mitochondrial respiratory chain Complex I [54] and down regulation of mitochondrial antioxidant defense [55] have been suggested to be biochemical hallmarks of seizure-induced neuronal cell death and epileptogenesis.

Tissue oxidative stress is involved in the inflammation-mediated multiple secondary injury cascades. Relationship between neuroinflammation and oxidative stress in hippocampus to seizure susceptibility, however, is controversial [15, 56]. Oral supplement with α-tocopherol, vitamin E with an antioxidant property, was reported to markedly reduce astrocytic and microglial activation, neuronal cell death and oxidative stress in the hippocampus [12]. Conversely, i.c.v. application of minocycline, a microglial inhibitor, significantly prevents oxidative damage to lipids and proteins in the hippocampus of rats subjected to systemic inflammation by cecal ligation and puncture [57]. As such, another important contribution of the present study is to unravel tissue oxidative stress as a downstream signaling to neuroinflammation in seizure susceptibility after peripheral inflammation. We found NS398 was effective to prevent the increases in NADPH oxidase expression and superoxide production (cf. Figs. 3 and 4); whereas tempol, at a dose that effectively diminished hippocampal oxidative stress, had no effect on the activation of microglia and production of cytokines (cf. Figs. 2 and 6) after peripheral inflammation. Additional signaling in exacerbating seizure susceptibility after peripheral inflammation can not be overlooked. In this regard, activation of the P2 class of ionotropic and metabotropic purinoceptors may mediate neural injury in the hippocampus after status epilepticus [58].

We realize that there are incurred limitations of the present study. First, a single dose of LPS was used in the present study to induce peripheral inflammation. We therefore do not know whether there is a threshold of severity for neuroinflammation and oxidative stress in the hippocampus on aggravation of seizure susceptibility. It is also unclear on impact of duration of the peripheral inflammation on seizure susceptibility. Second, the drug treatment regimen in this study was adopted to verify causal roles of neuroinflammation and oxidative stress in seizure susceptibility. Whether COX-2 and/or ROS inhibitors delivered after the establishment of neuroinflammation and oxidative stress in the hippocampus, conditions mimicking the clinical scenario, exert similar beneficial effects on seizure susceptibility remain to be confirmed.

## Conclusions

Our results provide evidence to support the notion that a COX-2-dependent activation of microglia and neuroinflammation, with subsequent oxidative stress in the hippocampus may lead to the aggravation of seizure susceptibility after peripheral inflammation. A wide range of diseases, including the inflammatory bowel disease [28], chronic asthma-associated airway inflammation [29], and HIV infection [59], manifest peripheral inflammatory. Our results may thus provide new information of the underlying mechanisms mediating the increase in seizure susceptibility under these disease conditions. In addition, these results provide evidence for the development of therapeutic interventions targeting neuroinflammation and brain oxidative stress for therapy of epilepsy.

## Abbreviations

COX-2: Cycloxygenase-2
CSF: Cerebrospinal fluid
Cu/ZnSOD: Copper/zinc superoxide dismutase
DG: Dentate gyrus
EPR: Electron paramagnetic resonance
GPx: Glutathione peroxidase
ICV: Intracerebroventricular
IL: Interleukin
IP: Intraperitoneal
KA: Kainic acid
LPS: Lipopolysaccharide
MnSOD: Manganese superoxide dismutase
NADPH: Nicotinamide adenine dinucleotide phosphate
PBS: Phosphate buffered saline
ROS: Reactive oxygen species
SOD: Superoxide dismutase
SOT3: Seizure onset time 3
TNF-α: Tissue necrosis factor-α
